# Ultra-Sensitive *CSF3R* Deep Sequencing in Patients With Severe Congenital Neutropenia

**DOI:** 10.3389/fimmu.2019.00116

**Published:** 2019-02-28

**Authors:** Maksim Klimiankou, Murat Uenalan, Siarhei Kandabarau, Rainer Nustede, Ingeborg Steiert, Sabine Mellor-Heineke, Cornelia Zeidler, Julia Skokowa, Karl Welte

**Affiliations:** ^1^Department of Hematology, Oncology, Immunology, Rheumatology and Pulmonology, University Hospital Tübingen, Tübingen, Germany; ^2^Department of Molecular Hematopoiesis, Hannover Medical School, Hannover, Germany; ^3^Department of Surgery, Children's Hospital, Hannover Medical School, Hannover, Germany; ^4^Department of Hematology, Oncology and Bone Marrow Transplantation, Hannover Medical School, Hannover, Germany; ^5^Department of Pediatric Hematology, Oncology and Bone Marrow Transplantation, University Hospital Tübingen, Tübingen, Germany

**Keywords:** severe congenital neutropenia, G-CSFR mutations, leukemogenesis, deep-sequencing, pre-leukemia

## Abstract

High frequency of acquired *CSF3R* (colony stimulating factor 3 receptor, granulocyte) mutations has been described in patients with severe congenital neutropenia (CN) at pre-leukemia stage and overt acute myeloid leukemia (AML) or myelodysplastic syndrome (MDS). Here, we report the establishment of an ultra-sensitive deep sequencing of a *CSF3R* segment encoding the intracellular “critical region” of the G-CSFR known to be mutated in CN-MDS/AML patients. Using this method, we achieved a mutant allele frequency (MAF) detection rate of 0.01%. We detected *CSF3R* mutations in CN patients with different genetic backgrounds, but not in patients with other types of bone marrow failure syndromes chronically treated with G-CSF (e.g., Shwachman-Diamond Syndrome). Comparison of *CSF3R* deep sequencing results of DNA and cDNA from the bone marrow and peripheral blood cells revealed the highest sensitivity of cDNA from the peripheral blood polymorphonuclear neutrophils. This approach enables the identification of low-frequency *CSF3R* mutant clones, increases sensitivity, and earlier detection of *CSF3R* mutations acquired during the course of leukemogenic evolution of pre-leukemia HSCs of CN patients. We suggest application of sequencing of the entire CSF3R gene at diagnosis to identify patients with inherited lost-of-function *CSF3R* mutations and annual ultra-deep sequencing of the critical region of *CSF3R* to monitor acquisition of *CSF3R* mutations.

## Introduction

Our current understanding of the pathological mechanisms responsible for severe congenital neutropenia (CN) suggests that it is a heterogeneous group of disorders with a common hematological and clinical phenotype characterized by a maturation arrest of myelopoiesis at the promyelocyte/myelocyte stage, resulting in a peripheral blood absolute neutrophil counts (ANCs) of <500 per microliter and early onset of bacterial infections. Although CN patients produce physiological amounts of G-CSF (granulocyte colony-stimulating factor), their myeloid precursor cells fail to differentiate normally into mature neutrophils; therefore, these patients require long-term treatment with pharmacological dosages of recombinant human G-CSF (rhG-CSF, Filgrastim). The number of G-CSF receptors (G-CSFRs) on myeloid precursor cells of CN patients is rather elevated suggesting that signaling pathways downstream of the G-CSFR are defective (1).

Over the last 10 years, remarkable progress has been achieved in identifying CN causing gene mutations. Mutations in the HAX1 (HCLS1-associated protein X-1) gene have been identified as the underlying genetic cause of the Kostmann syndrome, a subtype of autosomal recessive CN (2). Genetic analyses of autosomal dominant and sporadic cases of CN indicate that the majority of the ethnical European patient population harbor *ELANE* mutations encoding neutrophil elastase (elastase 2) (3). Interestingly, patients with cyclic neutropenia (CyN) also harbor mutations within the ELANE gene, even in the same nucleotide position (4, 5). In addition, mutations at a number of rarely affected genes, among them e.g., *G6PC3* (glucose 6 phosphatase, catalytic, 3) (6), *GFI1* (growth factor independent 1) (7), *TAZ* (tafazzin) (8), *WAS* (Wiskott-Aldrich syndrome) (9) and *JAGN1* (Jagunal Homolog 1) (10) have been identified in CN (11).

Various acquired point mutations in the intracellular domain of G-CSFR have been described. These mutations introduces premature stop codons, resulting in the truncated G-CSFR (12–19). Transfection of the mutated G-CSF receptor with truncated intracellular part into murine cell lines induced hyper-proliferative responses to G-CSF (12). These effects are also seen following co-expression of wild-type and truncated receptors; this so-called dominant-negative effect mirrors patient findings in cases where only one allele is mutated. Intriguingly, there is a high incidence of transformation to myelodysplasia (MDS) or acute myeloid leukemia (AML) in patients who harbor acquired *CSF3R* mutations, suggesting that these mutations are involved in the development of leukemia (19). Our hypothesis is that *CSF3R* mutations arise in hematopoietic stem cells by selective pressure and are present at a low level until this cell clone becomes dominant through the continuous rhG-CSF treatment and acquisition of additional mutations in a leukemia-associated genes, such as *RUNX1* (runt-related transcription factor 1) (20).

Several investigators reported the identification of acquired *CSF3R* mutations in CN patients. Mutation frequencies and detection methods varied dramatically between these studies (19, 21, 22). To date, many investigators have directly sequenced PCR fragments of the intracytoplasmic domain of the G-CSFR. Using the classic Sanger sequencing method, at least 15–20% of the cells investigated must harbor mutations to yield positive results; thus, this method does not allow detection of small sub-clones of *CSF3R*-mutated cells. We recently explored this problem in detail, reporting results obtained by sequencing multiple clones prepared from PCR fragments of the intracytoplasmic region of the G-CSFR from individual CN patients. Using up to 45 clones generated from mRNA of each patient and propagated as recombinant plasmids in *Escherichia coli*, we found that ~5% of RNA harboring *CSF3R* mutations could be detected (19).

Next-generation sequencing has significantly improved our ability to uncover genetic alterations in the genome. This novel approach allows the detection of low-abundance genetic aberrations, making it useful for the detection and monitoring of initial genetic lesions in AML at an early stage of leukemogenesis. Together with the sensitive detection of low-frequency minor mutant alleles, deep sequencing enables an accurate determination of allele frequencies.

We applied the sensitive deep sequencing of PCR products of the critical region of *CSF3R*, with more than 900,000 depth to detect the presence of *CSF3R* mutations during the course of leukemogenesis. We also investigated the influence of *CSF3R* mutations and single-nucleotide polymorphisms (SNPs) within *CSF3R* on G-CSF responsiveness in CN patients.

## Materials and Methods

### Patients and Controls

CN patients were diagnosed based on results of peripheral blood ANC values <0.5 × 10^9^/l within 3 months, examinations of bone marrow aspirates, a history of recurrent severe infections, and negative results for granulocyte-specific antibodies. All patients with a clinical diagnosis of CN were screened for mutations in *ELANE, HAX1, JAGN*1, and *G6PC3*. In the case of negative result, NGS bone marrow failure syndromes gene panel was performed.

In total, DNA from 54 CN, 17 CyN, 25 SDS, 19 CN-MDS/AML patients as well as 16 idiopatic neutropenia and 7 autoimmune neutropenia (AiN) patients was subjected of *CSF3R* DNA deep sequencing. Additionally, we sequenced groups of patients with clinical diagnoses unrelated to neutropenia, like pediatric *de novo* CML (*n* = 14), *de novo* AML (*n* = 10). We also used BM sample from healthy donors without (*n* = 11) or with (*n* = 2) rhG-CSFR treatment (Table S1). *CSF3R* deep sequencing of cDNA samples was performed using RNA isolated from 68 CN, 12 CyN, 13 SDS, 5 CN-MDS/AML, 15 idiopathic, and 2 AiN patients (Table 1). Nine patients with inherited syndromes associated with severe neutropenia (Cohen syndrome, WHIM syndrome, GSD-1b, Pearson syndrome, Barth syndrome, DBA, Hermansky-Pudlak syndrome) (Table 1) were also included in the study. On average more than 2 samples per CN patient were typically collected during 1–3 years of observation time and were available for *CSF3R* deep sequencing.

**Table 1:** Prevalence of *CSF3R* acquired mutations in studied groups using cDNA deep sequencing.

**Study groups**	**Number of patients, cDNA**	**Patients with acquired *CSF3R* mutations, cDNA**
**CN**:	68	32 (47.1%)
*ELANE-CN*	38	20 (52.6%)
*HAX1*-*CN*	20	9 (45%)
*G6PC3-CN*	3	1 (33.3%)
*WASP-CN*	1	1 (100%)
*JAGN1*-*CN*	4	1 (25 %)
*CSF3R-CN*	2	0
CN, genetically unclassified	28	16 (57.1%)
CyN	12	2 (16.7%)
Shwachman-Diamond syndrome (SDS)	13	0
CN-MDS/AML	5	4 (80%)
Idiopathic neutropenia	15	0
Autoimmune neutropenia	2	0
Others^*^	9	0
Total number of patients	152	54 (35.5%)

* *Cohen syndrome, WIHM syndrome, GSD-1b, Pearson syndrome, Barth syndrome, Hermansky-Pudlak syndrome, Diamond-Blackfan syndrome*.

Bone marrow and blood samples from patients were collected in association with an annual follow-up recommended by the Severe Chronic Neutropenia International Registry. Study approval was obtained from the Ethical Review Board of the Medical Faculty, University of Tübingen. Informed consent was collected in accordance with the Declaration of Helsinki.

### Nucleic Acid Isolation

Bone marrow mononuclear cells (BM-MNCs) and polymorphonuclear cells (BM-PMNs) as well as peripheral blood mononuclear cells (PB-MNCs) were isolated by Ficoll-Hypaque gradient centrifugation (Amersham Biosciences, UK). Peripheral blood polymorphonuclear cells (PB-PMNs) were isolated using Polymorphprep (AXIS-SHIELD PoC AS, Norway). CD34^+^ and CD33^+^ cells were separated from BM-MNCs by means of positive selection after incubation with corresponding MicroBead Kit (Miltenyi Biotec) according to a standard protocol. The purity of the isolated CD34^+^ and CD33^+^ cells was more than 80%, as evaluated by flow cytometry. RNA was extracted using an RNeasy Mini Kit (Qiagen, Germany) according the manufacturers' protocols. For DNA purification, DNeasy Blood and Tissue Kit (Qiagen, Germany) was used.

### Mutational Screening

Mutational screening of *ELANE, HAX1, JAGN1, G6PC3*, and *CSF3R* by Sanger sequencing was performed using a BigDye Terminator v3.1 Cycle Sequencing Kit on an ABI 3130 genetic analyzer (Applied Biosystems, USA) according to a standard sequencing protocol. Primer sequences are listed in Table S2. In cooperation with CeGaT (Germany) samples of CN patients negative for mutations in *ELANE, HAX1, JAGN1, G6PC3*, and *CSF3R* were subjected to gene panel sequencing approach for identification of potential mutations in 230 genes known to be associated with inherited hematological disorders.

### *CSF3R* DNA Deep Sequencing

DNA was extracted from patients and volunteers BM and PB as described above. Fragments of the CSF3R gene (4.4, 5.0, and 5.4 kb) were amplified by long-range PCR. PCR products were designed to cover all exons and introns of the CSF3R gene except intron 3 which was excluded because of low conservation among vertebrates and high enrichment on repetitive and low complexity DNA sequences. For all patients, the sequencing of all 3 long-range PCR product was performed only once. In all subsequent DNA samples 5.0 kb PCR product that includes a critical region of *CSF3R* was sequenced.

PCR products were used as a template for generating a fragment library with a Covaris S2 sonicator (Covaris, Inc., USA). Amplification of 4.4- and 5.0-kb fragments was performed using a Phusion High-Fidelity DNA Polymerase kit (Thermo Fisher Scientific Inc., USA) and a touchdown PCR approach (Tables S3, S5). The 5.4 kb fragment was amplified using an Advantage Genomic LA Polymerase Mix (Clontech Laboratories, Inc., CA, USA) (Tables S4, S5). Sonicated DNA fragments were size-selected using the Agencourt AMPure XP Bead Reagent (Beckman Coulter, Inc., USA). The 5500 SOLiD Fragment Library Enzyme Module (PN 4464413; Life Technologies, USA) was used to perform blunting/end polishing, dA-tailing, ligation of adaptors and barcodes to size-selected DNA. The Agencourt AMPure XP Reagent (Beckman Coulter, Inc., USA) was used for purification of the ligated DNA. After emulsion PCR and bead enrichment, 3′-modified beads were deposited onto a glass slide and sequenced by ligation using a 5500XL SOLiD Sequencer (Life Technologies, USA).

For the pre-processing, the input sequences underwent *De Novo* Error Correction for SOLiD data using the SOLiD Accuracy Enhancement Tool (Life Technologies, USA). Reads aligned by NovoAlignCS (novocraft.com) were collected as SAM files and then applied to our custom next-generation sequencing pipeline (V0.03), which includes the following workflow: SAMtools (23) for BAM file generation, Picard Tools for duplicate removal, Genome Analysis Toolkit for indel realignment (Broad Institute, USA), SAMtools for variant calling, and AnnTools (24) for annotating detected single-nucleotide variants. Only variants with a Phred quality score >20 were accepted.

For sequencing of the complete CSF3R gene, we chose a mutant allele frequency (MAF) of 0.02 as the default threshold for candidate calls and 0.007 if the number of reads exceeded more than 5000x depth of coverage at a mutation position.

### *CSF3R* cDNA Deep Sequencing

RNA was extracted from PB-MNCs, PB-PMNs, BM-MNCs, and BM-PMNs as described above. RNA purity and yield was measured on Nanodrop. Reverse transcription was performed by Omniscript RT Kit (Qiagen, Germany) according the manufacturers' protocol. Fragments of the CSF3R gene (236 and 198 bp) were amplified by PCR (Tables S6, S7). The PCR fragments were size-selected and purified using the Agencourt AMPure XP Bead Reagent (Beckman Coulter, USA). Before library preparation, PCR products were run on Bioanalyzer (Agilent, USA) to control purity.

Sequencing libraries were prepared according to 16S Metagenomic Sequencing Library Preparation Protocol from Illumina (Part#15044223Rev.B). Index PCR was performed using the Nextera XT Index Kit (Illumina, USA). For cleanup, AMPure XP Beads (Beckman, USA) were used. Libraries were quantified with Qubit HS Kit (Thermo Fisher Scientific, USA) and analyzed with Bioanalyser HS DNA Kit (Agilent, USA). Sequencing was performed on Illumina sequencer (NovaSeq 6000) with 2x100 bp sequencing mode using NovaSeq 5000/6000 S2 Flow Cell and appr. 0.6 Gb output per sample.

Demultiplexing, sequencing reads trimming, alignment, variant calling and annotation was done by NGS provider CeGaT (Germany). Demultiplexing of the sequencing reads was performed with Illumina bcl2fastq (2.19). Adapters were trimmed with Skewer (version 0.2.2) (25). Trimmed raw reads were aligned to the human reference genome (hg19) using the Burrows-Wheeler Aligner (BWA-mem version 0.7.17-cegat) (26). Low frequency variants were detected applying LoFreq (Version 2.1.2) (27). Mapping quality was determined by Burrows-Wheeler Aligner. Positions with coverage ≥ 10.000 and mapping quality of at least 15 (Phred-Score) were included for the variant calling used for the analysis. The quality of FASTQ files was analyzed with FastQC (version 0.11.5-cegat) (28). Plots were created using ggplot2 (29) in R (30). MAF of 0.001 was selected as the default threshold for candidate calls.

### Statistical Analysis

Statistical analyses were performed using R (30), Excel (Microsoft, USA) and Prism 7 version 7.03 (GraphPad Software, USA).

## Results

### Deep Sequencing of Whole *CSF3R* DNA as Well as of Critical *CSF3R* Region cDNA

**Figure 1:**
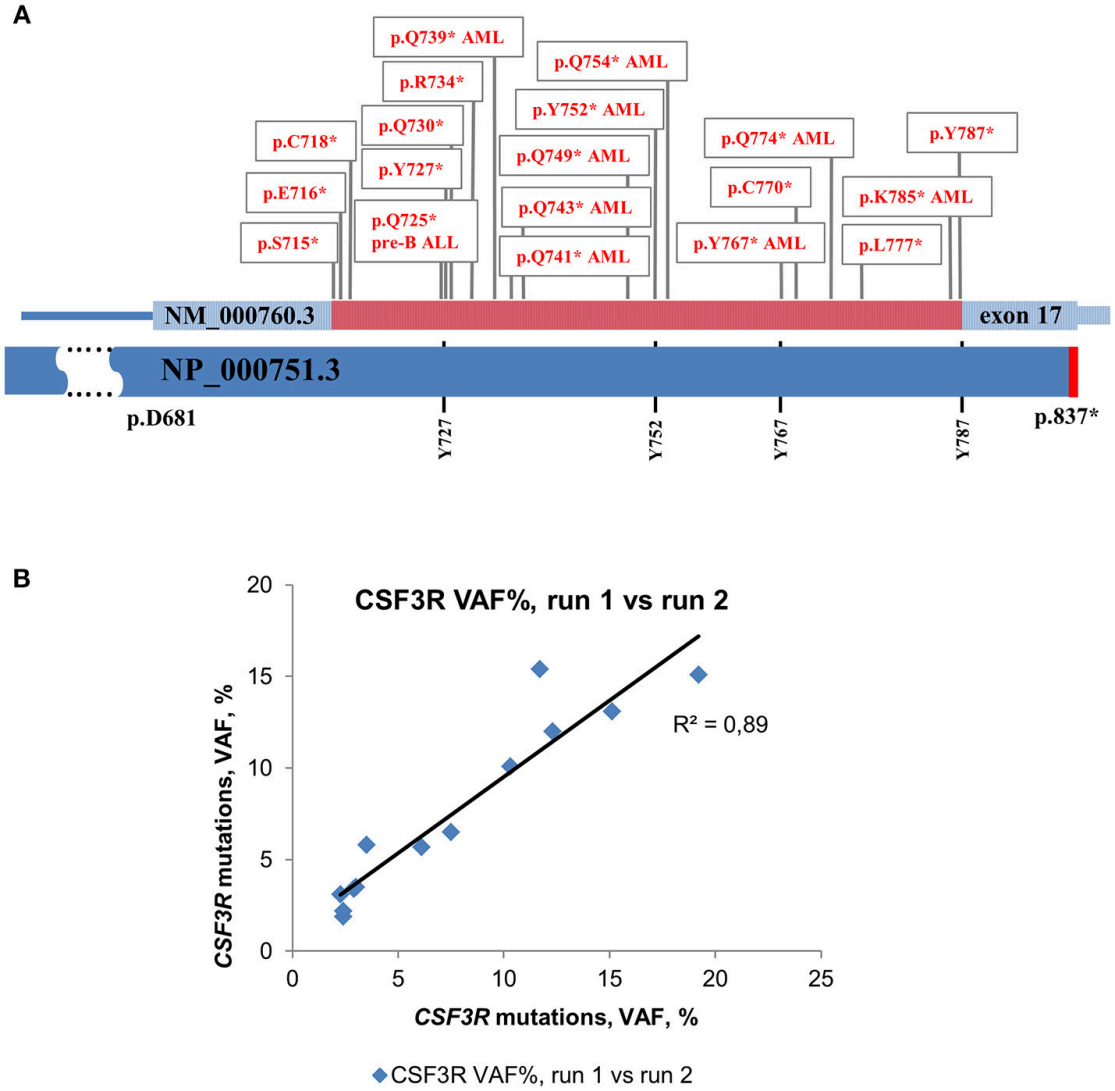
Distribution of acquired mutations in exon 17 of CSF3R in CN and CN-MDS/AML patients. Schematic representation of exon 17 of the CSF3R gene along with protein sequence (NP_000751.3) showing four conserved tyrosine residues. The amino acid positions corresponding to exon 17 are indicated. The intracellular critical region of *CSF3R* is marked in red. Amino acid (AA) positions of the CSF3R gene mutations are confined to the region between amino acid residues 715 and 787. Mutations in G-CSFR associated with leukemia and MDS in CN are denoted by “AML” or “pre-B ALL”, ^*^- stop codon. **(B)** Inter-run reproducibility between *CSF3R* DNA deep sequencing runs. Inter-run reproducibility was assessed by sequencing of 13 *CSF3R* mutations from 11 CN patients in two separate runs.

For deep sequencing analysis, we either sequenced PCR products of the whole CSF3R gene (except intron 3) or amplified cDNA of the intracellular region of G-CSFR that was reported to be mutated in CN and CN-MDS/AML patients. This region is lying between 715 and 787 amino acid positions (NP_000751.3) and included four conserved tyrosine residues, (Y727, Y752, Y767, and Y787) that act as docking sites for SH2 domain-containing proteins (Figure 1A) (31, 32).

The median age of patients at the time of sample collection was 13 years (25th percentile = 5.32, 75th percentile = 17.02) for cDNA samples and 12 years (25th percentile = 5.6, 75th percentile = 20.11) for DNA samples. In total, we sequenced 276 DNA samples and 289 cDNA samples. Patients included into the study are presented in Table 1 and Table S1.

In keeping with a previous report (19), we found that 73.7% (14/19) of CN patients at the AML or MDS stage acquired *CS3FR* mutations. We detected a *CSF3R* mutation in 1 out of 10 pediatric *de novo* AML patients and no mutations were found in 14 pediatric CML patients (Table S1).

The level of inter-run reproducibility was estimated by re-sequencing DNA samples from 11 CN patients, in which 13 *CSF3R* mutations were detected with MAF values from 2 to 19%. The re-sequencing results showed a high level of MAF concordance (*R*^2^ = 0.89) with consistent detection of all 13 mutations in a subsequent run (Figure 1B).

By sequencing of DNA samples, we identified *CSF3R* mutations in 20.4% (11/54) of patients in the genetically defined CN group, and in 28.5% (2/7) of genetically unclassified CN patients. The high frequency of *CSF3R* mutations was observed in CN patients harboring *HAX1* or *ELANE* mutations (30% (3/10) and 22.2% (8/36), respectively).

Notably, using deep sequencing of cDNA samples (Table 1), we detected *CSF3R* mutations in almost a half of CN patients (47.1% (32/68) in genetically defined CN group and 57.1% (16/28) in unclassified CN). We also detected *CSF3R* mutations in two CyN patients. No *CSF3R* mutations were found in other studied groups (Table 1). An eighty percent (4/5) of CN-MDS/AML patients were positive for *CSF3R* mutations (Table 1).

**Figure 2:**
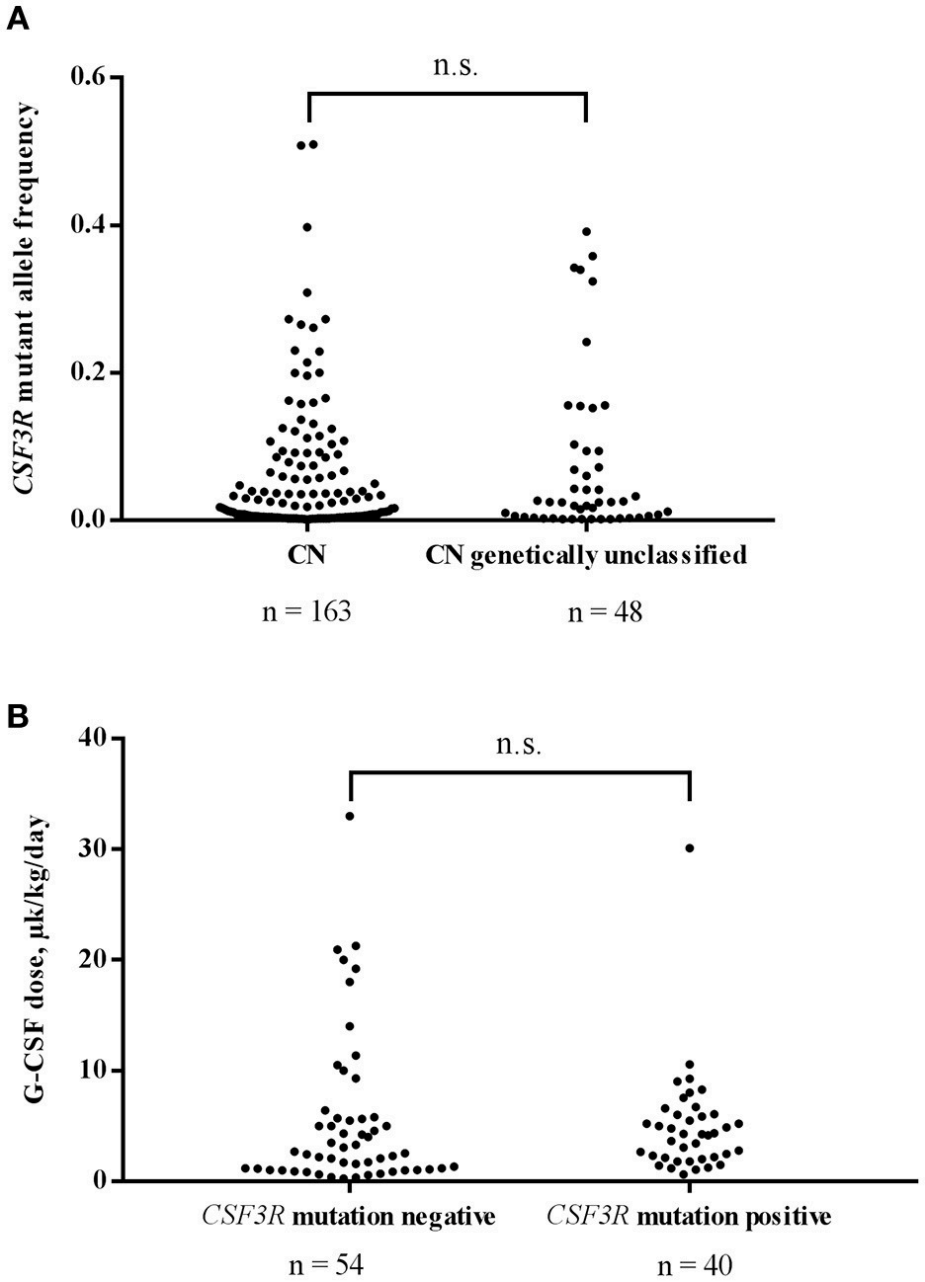
**(A)** Comparison of *CSF3R* mutant allele frequencies in CN and genetically unclassified CN patients. No significant difference in MAF of *CSF3R* mutations between CN (*n* = 163) and genetically unclassified CN (*n* = 48) groups was observed; n.s.- not significant. **(B)** The acquisition of *CSF3R* mutations is not associated with higher rhG-CSF doses. Patients were divided into 2 groups based on the presence (*n* = 40) or absence (*n* = 54) of *CSF3R* mutations detected by cDNA deep-sequencing. rhG-CSF doses at the time of sample collection are plotted for the both groups; n.s., not significant.

Using *CSF3R* cDNA deep sequencing, 163 mutant clones were identified in 77 samples of genetically defined CN patients with an average MAF of 0.051 and a median MAF 0.018. In genetically unclassified CN patients, we scored 48 *CSF3R* mutant clones in 28 positive samples with an average MAF of 0.074 and a median MAF of 0.025. No significant difference in MAF was observed between genetically defined and unclassified CN groups (Mann-Whitney *U*-test: *U* = 3,240, *P* > 0.99) (Figure 2A).

### No Correlation Between Acquisition of *CSF3R* Mutation and Therapeutic G-CSF Dose

For 102 patients, for whom information on rhG-CSF treatment was available, 96 received rhG-CSF treatment and were evaluated for association of acquired *CSF3R* mutation with the dose of rhG-CSF. We compared 40 patients harboring *CSF3R* mutations (12 *ELANE*-CN, 9 *HAX1*-CN, 1 *JAGN1*-CN, 3 CN-MDS/AML, 13 genetically unclassified CN and 2 CyN patients) with 54 patients without *CSF3R* mutations (10 *ELANE*-CN, 9 *HAX1*-CN, 2 *G6PC3*-CN, 1 *JAGN1*-CN, 1 CN-MDS/AML, 11 genetically unclassified CN, 7 CyN, 6 SDS, 1 idiopathic neutropenia patients as well as 6 patients with inherited syndromes associated with severe neutropenia (3 Cohen syndrome, 1 Barth syndrome, 1 WHIM, 1 GSDIb). Patients with germ-line *CSF3R* mutations (*n* = 2) were excluded from the analysis. Interestingly, we observed no difference in the rhG-CSF dose required to achieve more than 1,000 neutrophils/μl between patients with and without acquired *CSF3R* mutations (Mann-Whitney *U*-test: *U* = 890, *P* = 0.1473) (Figure 2B).

### Highest Sensitivity of Detection of *CSF3R* Mutations Was Observed in cDNA of PB-PMNs

**Figure 3:**
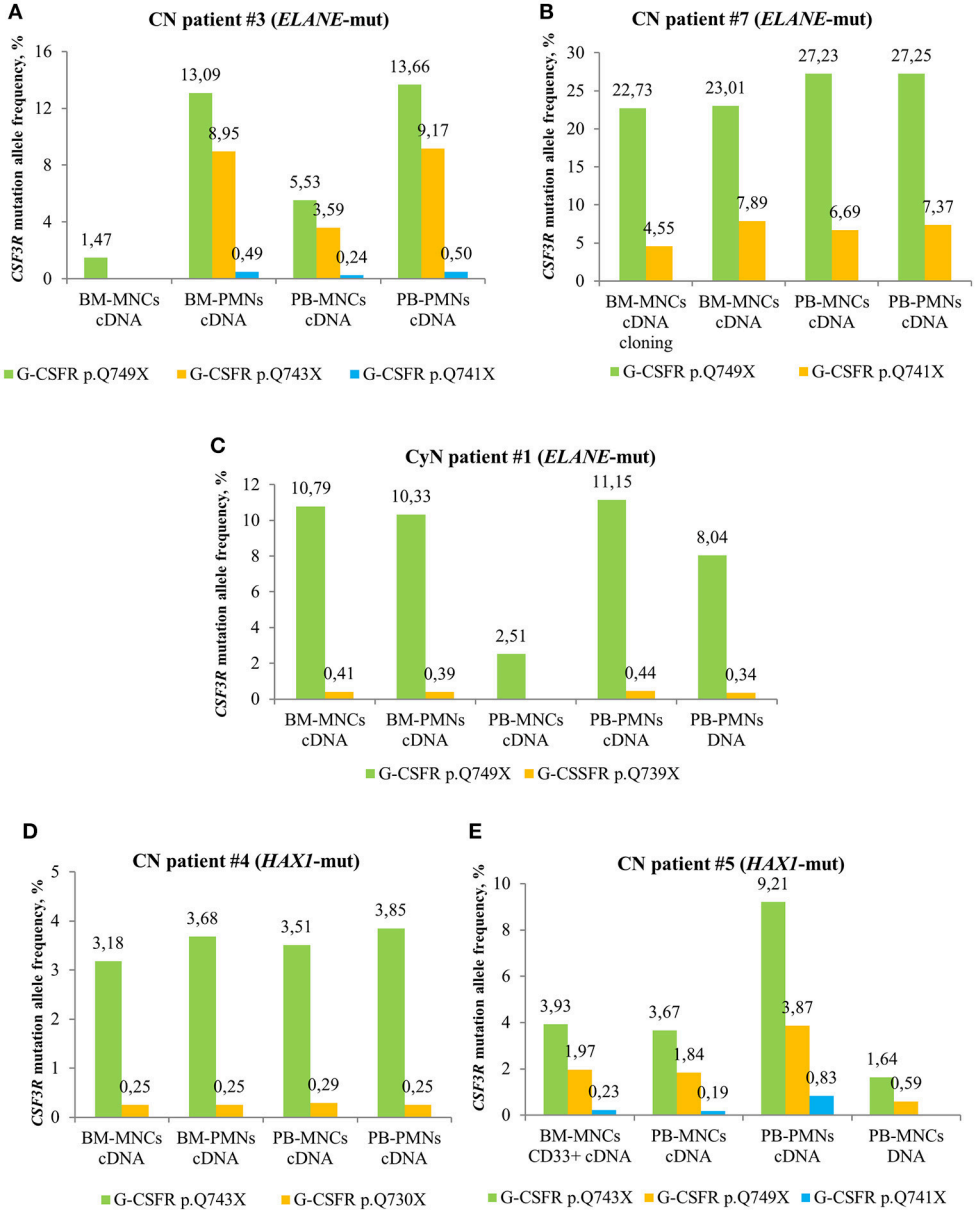
Comparison of deep sequencing results in different types of peripheral blood (PB) and bone marrow (BM) samples of CN and CyN patients. Frequency of *CSF3R* mutant clones in different types of PB and BM samples from *ELANE*-CN patients **(A,B)**, CyN patient **(C)** and *HAX1*-CN patients (**D,E**) was analyzed using cDNA deep sequencing, as described in Material and Methods section. The *CSF3R* mutant clones are indicated based on the relative amino acid positions of mutations.

We compared MAF of *CSF3R* mutations between cDNA and DNA isolated from different cell fractions of five CN patients and detected the highest MAF in PB-PMNs of all studied CN patients (Figures 3A–E), as compared to BM-MNCs, BM-PMNs, and PB-MNCs. As expected, the highest sensitivity was achieved by sequencing of cDNA samples. Based on these observations, we suggest sequencing cDNA of PB-PMNs, in order to achieve detection of low-frequency cell clones with mutated *CSF3R*.

### Time Course and Numbers of *CSF3R* Mutations in CN Patients

**Figure 4:**
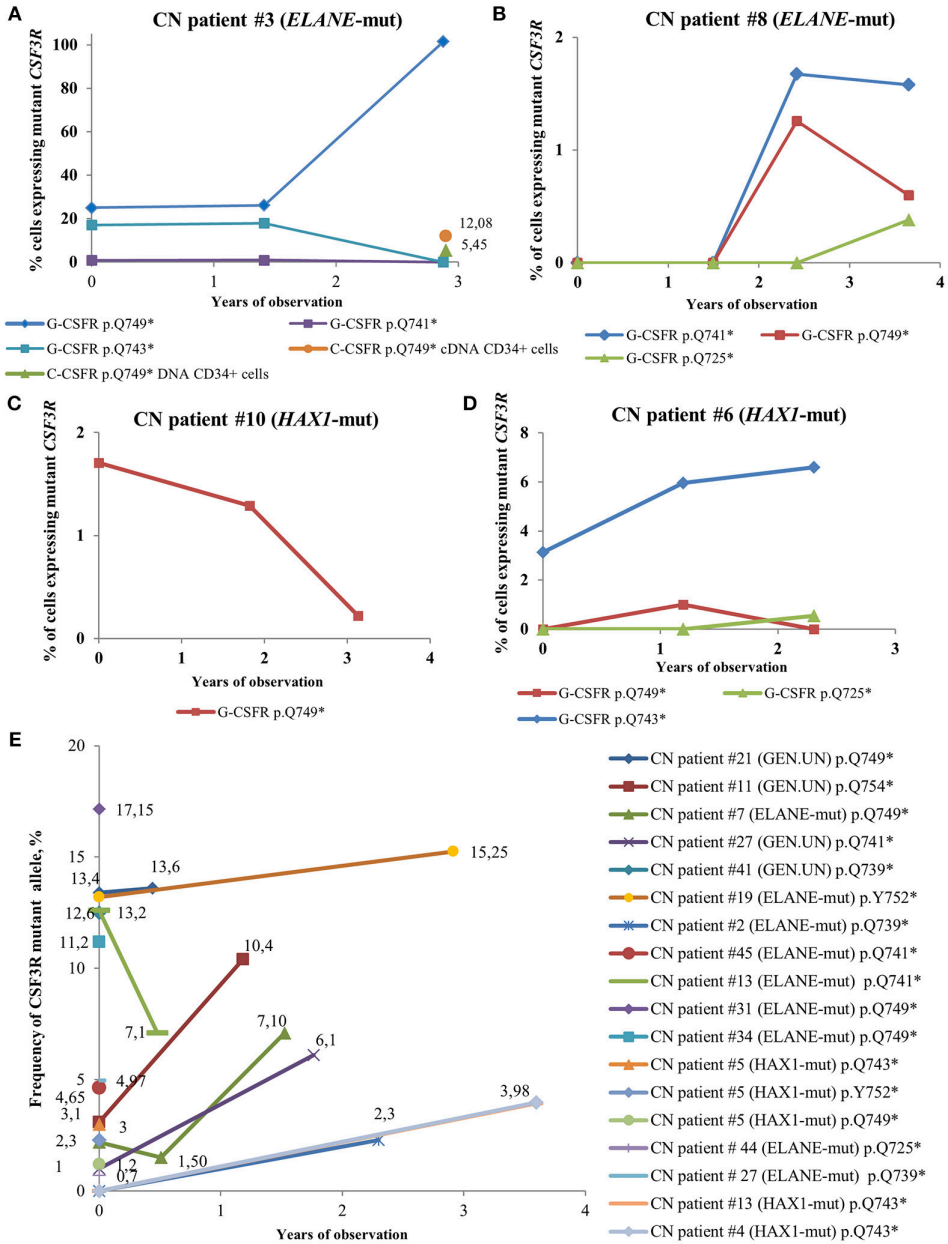
Time-course of *CSF3R* mutations in sequential samples from CN patients detected by cDNA and DNA deep sequencing. **(A–D)** The percentage of cells expressing mutant *CSF3R* at different time points (in years), starting from the date of the first *CSF3R* mutation analysis in a given patient, is plotted. All mutations are considered to be heterozygous. The *CSF3R* mutant clones are indicated based on the relative amino acid positions of mutation sites. The number of cells with *CSF3R* mutation is estimated to be twice the number of reads supporting a mutant allele, ^*^- stop codon. **(E)** Time-course of *CSF3R* mutations occurrence and frequency of mutant alleles in DNA samples from CN patients detected by *CSF3R* DNA deep sequencing. The frequency of mutant alleles at different time points (in years) in a given patient is plotted. The number of cells with *CSF3R* mutation is estimated to be twice the number of reads supporting a mutant allele, ^*^- stop codon.

In some CN patients, we identified multiple *CSF3R* mutations (Figure 4). Acquisition of multiple *CSF3R* mutations was not dependent on the type of CN-causing mutations. The percentage of cells expressing mutant *CSF3R* was varying during the years of observation with increasing MAF for some clones, but also decreasing MAF for other clones (Figure 4). Multiple *CSF3R* mutant clones were detected using sequencing of either cDNA (Figures 4A–D), or DNA (Figure 4E). Interestingly, in one *ELANE*-CN patient, all PB-PMNs acquired *CSF3R* mutation at the position p.Q749^*^ (Figure 4A). However, only a minor proportion of patient‘s bone marrow CD34^+^ hematopoietic stem cells had this mutation (5.45% of total CD34^+^ cells and 12.08% of G-CSFR expressing CD34^+^ cells) (Figure 4A).

For five CN patients with multiple *CSF3R* mutations, we investigated whether mutations were present in the same allele. Interestingly, all non-sense mutations in *CSF3R* were found to belong to different alleles (**data not shown)**.

### Low Frequency SNPs in the Coding Region of *CSF3R*

We next asked whether genetic polymorphisms in the CSF3R gene affect G-CSF response and, in turn, promote leukemogenic transformation in CN patients. We made a correspondence matrix with all identified SNPs for 83 patients with the available information on the therapeutic rhG-CSF dose. To select patients grouped by SNP combinations, we used hierarchical clustering with Jaccard distances and one-way ANOVA and did not find any significant differences in rhG-CSF dose between groups.

We analyzed the nucleotide variants identified in coding region of *CSF3R*. Among 598 genetic variants listed in public databases, 94 are missense and nearly 25% of them have minor allele frequency (MAF) <0.05. Six SNPs are associated with destructive effects (Table S8). In our patient's cohorts, we identified 13 SNPs with MAF <0.05 and 3 novel variants (Tables S9, S10). The most frequently detected variants with low MAF were p.D510H (9/173) and p.D320N (9/173) (Table S10). The p.D510H SNP was predicted to have damaging effects on protein functions by 4 out of 6 *in silico* prediction algorithms (Table S9). The therapeutic rhG-CSF doses in CN patients carrying this SNP varied between 0.2 and 11.3 μg/kg/day. The p.D320N SNP, which is located in the conserved cytokine receptor homology domain, was predicted to be benign by all six prediction algorithms. Interestingly, in one CyN patient we detected a novel nucleotide polymorphism at p.M222T in the fibronectin type III like domain of the G-CSF receptor which was predicted to have the severe effects on protein function by 4 different algorithms (Table S9).

We identified one CN patient with p.R311C SNP, that have a novel variant, p.Q114^*^ in *CSF3R* and no mutations in *ELANE, HAX1, G6PC3*, and *GFI1*. Of all identified rare SNPs, p.R311C had the most severe effects on protein structure and function (Table S9). CN patient with p.R311C and p.Q114^*^ SNPs is responding to high dose of rhG-CSF (median dose 40 μg/kg/day) and the role of these variants as potential causative factors for neutropenia cannot be excluded. Additionally, in one CN patient with no response to G-CSF bi-allelic loss-of-function *CSF3R* mutations: the heterozygous SNP p.W547^*^ and a novel mutation at the 3′ splice-acceptor site of intron 8 c.998-2A>T were detected (Table S10) (33). Except one CN patient with p.D510H SNP, no other G-CSF poor or non-responders had genetic alterations in the CSF3R gene. Four rare SNPs (p.M231T, p.Q346R, p.E405K, and p.A750T) were identified in CN patients who required a G-CSF dose <2 μg/kg/day, and one SNP (p.E149D) was detected in a CN patient who was not treated with G-CSF. Intriguingly, 1 CML and 1 CN patients carried a p.E808K SNP, which is known to correlate with high-risk of MDS development (34).

## Discussion

We demonstrated for the first time the establishment of sensitive NGS technology that allows identification of low-frequency cell clones with acquired *CSF3R* mutations across the CSF3R gene in a large group of patients with different types of CN. We found, that ultra-deep sequencing of cDNA markedly increased the sensitivity and identification of *CSF3R* mutant clones with MAF of 0.001. Much higher sensitivity of *CSF3R* cDNA over DNA deep sequencing can be explained by in average 900-fold increase of sequencing depth and selection of G-CSFR expressing cell population. We speculate that further increase of the sensitivity may lead to identification of *CSF3R* mutations virtually in all *ELANE*-CN and *HAX1*-CN patients. Our findings also suggest, that HSC clones with acquired *CSF3R* mutations are not leukemic, but pre-leukemic clones with an increased susceptibility to secondary leukemogenic events (e.g., *RUNX1* mutation, trisomy 21) and overt MDS or AML. We detected no correlation between the presence of *CSF3R* mutations and disease severity or G-CSF dose required to achieve sufficient neutrophil counts.

Previous studies have reported contradictory findings on the safety of G-CSF treatment (35–37). This is an important issue, since G-CSF is used not only to treat different types of CN, but is also applied for chemotherapy-induced neutropenia and to induce mobilization of CD34^+^ stem and progenitor cells for autologous or allogeneic transplantation. To determine whether long-term G-CSF treatment *per se* induces acquisition of *CSF3R* mutations or is a CN-specific phenomenon, we examined the association between G-CSF treatment and acquisition of *CSF3R* mutations. There was no difference in therapeutic G-CSF dose between patients with or without acquired *CSF3R* mutations. Intriguingly, we detected no acquired *CSF3R* mutations in a large group of SDS patients who were on long-term G-CSF treatment. This is in accordance with the report of Xia et al. who demonstrated that clonal hematopoiesis due to mutations in *TP53*, but not *CSF3R* was present in patients with SDS but was not detected in healthy controls or patients with CN (38). We also found no acquired *CSF3R* mutations in G-CSF treated healthy individuals, although the group was small and G-CSF treatment was short-term. Acquired *CSF3R* mutations were detected exclusively in CN patients. These observations strongly argue for the safety of G-CSF therapy. Most likely, intracellular molecular defects specific for disturbed granulopoiesis in CN patients (39) and not G-CSF therapy *per se*, is responsible for the acquisition of *CSF3R* mutations in some HSC clones and clonal overgrowth of these clones.

We have shown in this report and in earlier studies (19) that ~80% of CN patients who developed leukemia harbor acquired *CSF3R* mutations, and that all leukemic cells in these patients are affected by these mutations. Our *CSF3R* sequencing approach allows direct evaluation of the frequency of *CSF3R* mutant cells, which is not possible with Sanger sequencing and possible to a certain extent with Sanger sequencing of cloned PCR products. The evidence of clonal hematopoiesis in CN patients without signs of malignant transformation suggest that secondary promoting mutations (e.g., in *RUNX1, ASXL1, SUZ12*, or *EP300*) (20, 40) are necessary to transform the *CSF3R* mutant clone in bone marrow. By annual monitoring of *CSF3R* mutations in CN patients using the deep sequencing we were able to trace pre-leukemic clones over a long period of time. Sequencing additional genes known to be associated with leukemogenesis in CN (e.g., *RUNX1*) might help to identify early stages of malignant transformation and could have a decisive role in guiding clinical decisions.

The time between acquisition of *CSF3R* mutations and overt leukemia vary substantial, some patients carry *CSF3R* mutations for more than 10 years. The persistence of mutated clones for years in CN patients without overt leukemia is intriguing. Interestingly, sequential analyses of the same patients at multiple time points led to increase in detection of *CSF3R* mutations to 34% (19). In our study, we found that almost 50% of CN patients acquired *CSF3R* mutations. Based on these data, we hypothesize that a sub-clone of pre-leukemic cells carrying *CSF3R* mutations at a frequency below our detection limit may be present for a long time and evolves to a frequency detectable by the deep-sequencing method in response to e.g., ER stress through unfolded protein response (UPR) caused by germline mutations. This is also supported by the fact, that some clones harboring *CSF3R* mutations evolved and disappeared again below the limit of detection but are most likely not absent completely (19, 20).

Interestingly, acquisition of *CSF3R* mutations in CN patients was independent of the inherited mutational status (Table 1). These data are consistent with our previous observations that components of the G-CSF receptor signaling such as the transcription factors LEF-1 and C/EBPα are severely diminished in myeloid progenitor cells of CN patients harboring *ELANE* or *HAX1* mutations (41).

All acquired mutations identified by us in CN and CN-MDS/AML patients using deep-sequencing technology were located in region encoding the critical intra-cytoplasmic domain of the G-CSFR which is known as the only region mutated in CN-MDS/AML patients (19, 42). This region has four conserved tyrosine residues essential for appropriate activation and suppression of G-CSFR signaling by STAT3 (signal transducer and activator of transcription 3) and SOCS3 (suppressor of cytokine signaling 3), respectively (31). The absence of these conserved tyrosine residues in cells expressing truncated G-CSFR might lead to a strong proliferative advantage (43–45).

We and others have identified CN patients with germ-line bi-allelic *CSF3R* mutations in the extracellular domain of the receptor that lead to G-CSF non-responsiveness (33, 46). We suggest sequencing the entire coding sequence of *CSF3R* in G-CSF “non-responders.” All CN patients regardless of their mutation status, except SDS patients, should undergo annual deep sequencing of the critical intra-cellular region of *CSF3R*.

Interestingly, in one of ten pediatric *de novo* AML patients, we also identified a non-sense *CSF3R* mutation that was located in the critical region of the G-CSFR that affected one of four essential tyrosine residues (Y787). No mutations were observed in pediatric CML patients; however, one CML patient carried the rare SNP, p.E808K, which is known to be correlated with the development of high-risk MDS (34). *CSF3R* mutations in *de novo* AML patients are very rare, and mutations leading to the absence of functionally important tyrosine residues had not been previously described (47–49). Further studies of larger cohorts of AML and CML patients will be required to evaluate the role of *CSF3R* mutations and rare SNPs in the development of *de novo* AML and CML.

Up to now, the role of SNPs in *CSF3R* in CN patients was unclear. By sequencing the entire CSF3R gene, we were able to identify and analyze rare SNPs. We identified 13 dbSNPs with MAF < 0.05, 2 novel variants in the coding region and one variant at an intron-exon junction (Table S10). However, there were no clear correlations between the presence of distinct rare SNPs and type of neutropenia, G-CSF dose requirement, leukemia progression, or disease severity. Only 2 of 10 CN patients with a poor or no G-CSF response had a rare SNP, effectively excluding intrinsic defects in *CSF3R* as a main cause of poor/non-responsiveness, at least in this studied group.

In summary, we have reported the establishment of sensitive *CSF3R* deep sequencing for assessing *CSF3R* mutation status in neutropenia patients. We suggest sequencing of cDNA of PB-PMNs to monitor hematopoietic clones that acquired *CSF3R* mutations over years. We found that acquisition of *CSF3R* mutations is a CN-specific phenomenon and is not present in patients with other types of neutropenia chronically treated with rhG-CSF.

## Author Contributions

JS and KW designed and supervised the study. MK and IS performed the main experiments. MU, SK, and MK analyzed deep-seq data. RN helped with sample preparation for NGS. JS, KW, and MK analyzed the data and wrote the manuscript. CZ and SM-H provided patient's material and clinical data.

### Conflict of Interest Statement

The authors declare that the research was conducted in the absence of any commercial or financial relationships that could be construed as a potential conflict of interest.
