# Supplementary material for: Ultra-Sensitive CSF3R Deep Sequencing in Patients With Severe Congenital Neutropenia
Source: Front Immunol. 2019 Feb 28;10:116. doi: 10.3389/fimmu.2019.00116 (PMC6413711; doi:10.3389/fimmu.2019.00116)
Supplement: Supplementary file 1 [file Data_Sheet_1.doc]

**Supplementary Table 1. Prevalence of *CSF3R* acquired mutations in studied groups using DNA deep sequencing.**

| Study groups | Number of patients, DNA | Patients with acquired *CSF3R* mutations, DNA |
| --- | --- | --- |
| **CN:** | **54** | **11 (20,4%)** |
| *ELANE*-CN | 36 | 8 (22,2%) |
| *HAX1*-CN | 10 | 3 (30%) |
| *G6PC3*-CN | 5 | 0 |
| *JAGN1*-CN | 1 | 0 |
| *CSF3R*-CN | 2 | 0 |
| **CN, genetically unclassified** | **7** | 2 (28,5%) |
| **CyN** | **17** | 1 (5,9%) |
| **Shwachman-Diamond Syndrome (SDS)** | **25** | 0 |
| **CN-MDS/AML** | **19** | **14 (73,7%)** |
| **Idiopatic neutropenia** | **16** | 0 |
| **Autoimmune neutropenia** | **7** | 0 |
| **Others*** | **4** | 0 |
| ***de novo* AML** | **10** | **1 (10%)** |
| **CML** | **14** | 0 |
| **Total number of patients** | **173** | **29 (16,7%)** |
| **Healthy individuals:** | **13** | 0 |
| without rhG-CSF treatment | 11 | 0 |
| treated with rhG-CSF | 2 | 0 |

*WHIM syndrome, GSD-1b, Pearson syndrome

**Supplementary Table 2**. Primers to amplify coding exons of *ELANE*, *HAX1*, *JAGN1*, *G6PC3*, and *CSF3R*.

|  | **Primer name** | **Primer sequence** | **Fragment size, bp** |
| --- | --- | --- | --- |
| 1 | ELANE.ex1F | GCAATGCAACGGCCTCCCA | 321 |
| 2 | ELANE.ex1.R | CAGAACCTCAGTCTCTTCTG |
| 3 | ELANE.ex2.F | GGCTCCTTGGCAGGCACTCA | 270 |
| 4 | ELANE.ex2.R | CACCTCACAGACCGGGACGC |
| 5 | ELANE.ex3.F | TCGAGCACCTTCGCCCTC | 400 |
| 6 | ELANE.ex3.R | CACAGAGGTGCAGACCGAG |
| 7 | ELANE.ex4.F | CGCCCTGAGCCTTGGTGACG | 450 |
| 8 | ELANE.ex4.R | AGCCACGGTGCCTGTTGCTG |
| 9 | ELANE.ex5.F | CAGCCCGGACTGCAGCAACA | 350 |
| 10 | ELANE.ex5.R | GACCCGGGCAGCCCTTCTCA |
| 11 | HAX1.ex1/2A.F | TGCTTTCCGGTAGCGTGGGC | 207 |
| 12 | HAX1.ex1/2A.R | ACGACCCCCACCCTTGTCCG |
| 13 | HAX1.ex2B.F | CCATGAGTTGATTTAATGGCTT | 442 |
| 14 | HAX1.ex2B.R | GAAATCATCAGTCTTCTCACACT |
| 15 | HAX1.ex3.F | GAGATTAATAGAGCCCAAGTC | 291 |
| 16 | HAX1.ex3.R | GGTCTCTCACAAGCTCTCAC |
| 17 | HAX1.ex4.F | GAATATATCAGTTCAGGAAGTC | 311 |
| 18 | HAX1.ex4.R | CACACAGAACTTCAGAAGAATA |
| 19 | HAX1.ex5.F | CTGAAACAGGGATCTGTGTAA | 214 |
| 20 | HAX1.ex5.R | CCAAGGAATCTTGAGATGAAC |
| 21 | HAX1.ex6.F | TATTCTTCTGAAGTTCTGTGTG | 292 |
| 22 | HAX1.ex6.R | TTACACAGATCCCTGTTTCAG |
| 23 | HAX1.ex7.F | GTTCATCTCAAGATTCCTTGG | 441 |
| 24 | HAX1.ex7.R | CAAGATAATTTCGGAAGTGTTG |
| 25 | G6PC3.ex1.F | TACCGGCTGGAGGCCGGTCTTG | 515 |
| 26 | G6PC3.ex1.R | GGGTGGGAAGGCCCTTGAGACA |
| 27 | G6PC3.ex2.F | ACCCCTCAGGCCCTCCAGAGTA | 294 |
| 28 | G6PC3.ex2.R | TTGGGCCACCCAGGCTGAAAA |
| 29 | G6PC3.ex3.F | AAAAGGCCTCAGAGCAGAGCGA | 389 |
| 30 | G6PC3.ex3.R | GCCTCCTGCAGCACCCAGGTA |
| 31 | G6PC3.ex4.F | ATTGGGAGCAGGGGTGATGGCA | 476 |
| 32 | G6PC3.ex4.R | ATATGACCCTCCCCACCCCGA |
| 33 | G6PC3.ex5.F | GCTCCCAGCTTCTGTAGAGCCC | 463 |
| 34 | G6PC3.ex5.R | GCTCTTGGCCATCTGCCCTTGG |
| 35 | G6PC3.ex6.1.F | GAACATGGGAGTGGGCCCCAAG | 438 |
| 36 | G6PC3.ex6.1.R | CGGTGCTTCCTGGGCACTGAAC |
| 37 | G6PC3.ex6.2.F | GTCGGGCACAGCTGGGAAATGG | 629 |
| 38 | G6PC3.ex6.2.R | CAGGGCTGGAGATGGCACAGA |
| 39 | JAGN1.ex1.F | CCGGAAGTTCTCTTCACGGA | 510 |
| 40 | JAGN1.ex1.R | TTCGAAGGTGATACAGCGGG |
| 41 | JAGN1.ex2.F | GCCAGGCAGAGTTGATTCCT | 889 |
| 42 | JAGN1.ex2.R | CCCAGTTCTAGCCAACCAGG |
| 43 | G-CSFR.ex17.1F | ATGGCATGTGTCAGGCATGT | 450 |
| 44 | G-CSFR.ex17.1R | AGTCACAGCGGAGATAGTGC |
| 45 | G-CSFR.ex17.2F | CACAGTGCTGGAGGAGGATG | 577 |
| 46 | G-CSFR.ex17.2R | TGGAGATTGGGAGGAGAGGG |

**Supplementary Table 3.** PCR cycling conditions for amplification of 4.4 and 5.0 kb DNA fragments of

*CSF3R*.

| PCR step | Temperature | Time | Number of cycles |
| --- | --- | --- | --- |
| Initial denaturation | 98^0^C | 30sec | 1cycle |
| Denaturation | 98^0^C | 10sec | 10cycles |
| Annealing | 73^0^C (decrement – 0.5^0^C/cycle) | 30sec |
| Extension | 72^0^C | 3:20min |
| Denaturation | 98^0^C | 10sec | 25cycles |
| Annealing | 68^0^C | 30sec |
| Extension | 72^0^C | 3:20min |
| Final extension | 72^0^C | 10min | 1 cycle |
| Hold | 5^0^C | ∞ | - |

**Supplementary Table 4. PCR cycling conditions for amplification of 5.4 kb DNA fragment of *CSF3R*.**

| PCR step | Temperature | Time | Number of cycles |
| --- | --- | --- | --- |
| Initial denaturation | 94^0^C | 1min | 1cycle |
| Denaturation | 98^0^C | 10sec | 35 cycles |
| Annealing/extension | 68^0^C | 7min |
| Final extension | 72^0^C | 10min | 1 cycle |
| Hold | 5^0^C | ∞ | - |

**Supplementary Table 5.** Primers to amplify DNA of *CSF3R* gene.

|  | Name | Primer sequence | Nts | Fragment size, bp |
| --- | --- | --- | --- | --- |
| 1 | CSF3R_4.4kb_F | TCAGACTTGAATCTTGTGGGATTCTCTGGG | 30 | 4405 |
| 2 | CSF3R_4.4kb_R | TATGTTAAGGACAGTCTGCTGGTCTGTTGG | 30 |
| 3 | CSF3R_5.0kb_F | AGCTGCCACCACCCACTGCTT | 21 | 4991 |
| 4 | CSF3R_5.0kb_R | CCGTGTCCCACCGCACTGTC | 20 |
| 5 | CSF3R_5.4kb_F | GGGAAAGCAGTGGGTGGTGGC | 21 | 5441 |
| 6 | CSF3R_5.4kb_R | GCTTGCACCATCCTTGGGGAGG | 22 |

**Supplementary Table 6.** PCR cycling conditions for amplification of cDNA fragments of *CSF3R* gene.

**Temperature profile for amplification of CSF3R.p01.deep PCR product**

| PCR step | Temperature | Time | Number of cycles |
| --- | --- | --- | --- |
| Initial denaturation | 98^0^C | 30sec | 1cycle |
| Denaturation | 98^0^C | 10sec | 35cycles |
| Annealing | 78^0^C | 10sec |
| Extension | 72^0^C | 7sec |
| Final extension | 72^0^C | 5min | 1 cycle |
| Hold | 8^0^C | ∞ | - |

**Temperature profile for amplification of CSF3R.p02.deep PCR product**

| PCR step | Temperature | Time | Number of cycles |
| --- | --- | --- | --- |
| Initial denaturation | 98^0^C | 30sec | 1cycle |
| Denaturation | 98^0^C | 10sec | 35cycles |
| Annealing | 65^0^C | 10sec |
| Extension | 72^0^C | 5sec |
| Final extension | 72^0^C | 5min | 1 cycle |
| Hold | 8^0^C | ∞ | - |

**Supplementary Table 7.** Primers to amplify cDNA of the *CSF3R* gene.

|  | Name | Primer sequence | Nts | Fragment size, bp |
| --- | --- | --- | --- | --- |
| 1 | CSF3R.p01.deep.F | TCGTCGGCAGCGTCAGATGTGTATAAGAGACAGCCGGTGCCCTGGGAG | 48 | 236 |
| 2 | CSF3R.p01.deep.R | GTCTCGTGGGCTCGGAGATGTGTATAAGAGACAGGGCTTGTGGGGCTGC | 49 |
| 3 | CSF3R.p02.deep.F | TCGTCGGCAGCGTCAGATGTGTATAAGAGACAGGGGCAGCCCCACAAGC | 49 | 198 |
| 4 | CSF3R.p02.deep.R | GTCTCGTGGGCTCGGAGATGTGTATAAGAGACAGGGGTCCCCAAGGGGCT | 50 |

**Supplementary Table 8.** dbSNPs in the G-CSF receptor gene.

| dbSNP 142 (Homo sapiens Annotation Release 106), *CSF3R* gene | Variant type | Molecular consequence | Number of variants | Minor allele frequency (MAF), 1000 Genomes Project | | | | |
| --- | --- | --- | --- | --- | --- | --- | --- | --- |
|  |  |  |  | <0.005 | 0.005-0.01 | 0.01-0.05 | >=0.05 | Not specified |
|  | Single nucleotide variant | Missense | 94 | 24 | 4 | 4 | 1 | 61 |
|  |  | Nonsense | 2 | 1 | - | - | - | 1 |
|  |  | Synonymous | 60 | 14 | 2 | 1 | 2 | 41 |
|  |  | Splice donor | 1 | - | - | - | - | 1 |
|  |  | Intron | 358 | 133 | 24 | 39 | 28 | 134 |
|  |  | 5 prime UTR | 46 | 18 | - | 2 | 1 | 25 |
|  |  | 3 primer UTR | 13 | 4 | - | - | - | 9 |
|  |  | 500bp downstream | 8 | 2 | - | - | 1 | 5 |
|  |  | 2kb upstream | 79 | 33 | 4 | 6 | 7 | 29 |
|  | Deletion | Frameshift | 1 | - | - | - | - | 1 |
|  |  | Intron | 18 | - | - | 1 | 3 | 14 |
|  |  | 2kb upstream | 2 | - | - | - | - | 2 |
|  | Insertion | Frameshift | 2 | - | - | - | - | 2 |
|  |  | Intron | 14 | - | - | - | 2 | 12 |
|  |  | 5 prime UTR | 1 | - | - | - | - | 1 |
|  |  | 500bp downstream | 1 | - | - | - | - | 1 |
|  |  | 2kb upstream | 4 | - | 1 | - | - | 3 |
| Total number of SNPs 598 | | | | | | | | |

The data retrieved from dbSNP 142 (Homo sapiens Annotation Release 106), based on the GRCh38 and GRCh37.p13 assemblies and are available on <http://www.ncbi.nlm.nih.gov/variation>.

**Supplementary table 9. Prediction of possible impact of low frequency SNPs in coding regions of the G-CSFR gene (general MAF= < 0.05) detected in studied patient’s groups on its structure and functions.**

| **Amino acid exchange** | **Protein RefSeq** | **ID dbSNP** | **PROVEAN** | **PROVEAN prediction** | **SIFT** | **SIFT prediction** | **Mutation**  **Taster** | **Mutation Assessor** | **FATHMM** | **PolyPhen-2 HumVar** | **PolyPhen-2 HumDiv** | **Total score** |
| --- | --- | --- | --- | --- | --- | --- | --- | --- | --- | --- | --- | --- |
| p.A750T | NP_758519.1 | rs142939584 | -0.14 | neutral | 0.022 | damaging | polymorphism | N/A | N/A | benign | benign | 1/4 |
| p.D320N | NP_000751.1 | rs3918018 | -1.73 | neutral | 0.301 | tolerated | polymorphism | neutral | TOLERATED | benign | benign | 0/6 |
| p.D510H | NP_000751.1 | rs3917991 | -4.12 | deleterious | 0.041 | damaging | polymorphism | medium | TOLERATED | possibly damaging | probably damaging | 3.5/6 |
| p.E149D | NP_000751.1 | rs139332126 | -0.34 | neutral | 1 | tolerated | polymorphism | neutral | TOLERATED | benign | benign | 0/6 |
| p.E405K | NP_000751.1 | rs3918019 | -0.65 | neutral | 0.378 | tolerated | polymorphism | low | TOLERATED | benign | possibly damaging | 0.5/6 |
| p.E808K | NP_000751.1 | rs146617729 | -1.12 | neutral | 0.057 | tolerated | desease causing | low | TOLERATED | possibly damaging | probably damaging | 2/6 |
| p.E835K | [NP_724781.1](http://www.ncbi.nlm.nih.gov/protein/24496783) | rs146617729 | -1.02 | neutral | 0.024 | damaging | desease causing | low | TOLERATED | possibly damaging | probably damaging | 3/6 |
| p.G683R | NP_724781.1 | rs3918001 | -0.22 | neutral | 0.424 | tolerated | polymorphism | N/A | TOLERATED | benign | benign | 0/6 |
| p.M222T | NP_000751.1 | novel | -3.22 | deleterious | 0.008 | damaging | polymorphism | medium | TOLERATED | probably damaging | probably damaging | 3.5/6 |
| p.M231T | NP_000751.1 | rs3917973 | 0.27 | neutral | 0.779 | tolerated | polymorphism | neutral | TOLERATED | benign | benign | 0/6 |
| p.M696T | NP_724781.1 | rs148916169 | 0.01 | neutral | 0.056 | tolerated | polymorphism | N/A | TOLERATED | benign | benign | 0/6 |
| p.Q346R | NP_000751.1 | rs3917974 | -0.93 | neutral | 0.228 | tolerated | polymorphism | low | TOLERATED | benign | benign | 0/6 |
| p.R440Q | NP_000751.1 | rs3918020 | 0.16 | neutral | 0.401 | tolerated | polymorphism | neutral | TOLERATED | benign | benign | 0/6 |
| p.R311C | NP_000751.1 | rs201890478 | -4.11 | deleterious | 0.054 | damaging | desease causing | medium | TOLERATED | probably damaging | probably damaging | 4.5/6 |

For all 14 low frequency dsSNPs (MAF< ~0.05) and novel SNP possible impact on G-CSFR structure and function was estimated using 6 different prediction algorithms (PROVEAN, SIFT, Mutation Tester, Mutation Assessor, FATHMM and PolyPhen-2). PROVEAN score has predefined threshold -2.28. If the PROVEAN score for variant is above the threshold, the variant is predicted to have a "neutral/tolerated"effect, if it is below, the variant is predicted to have a "deleterious/damaging" effect. SIFT score ranges from 0 to 1. The variant is predicted damaging is the score is <= 0.05, and tolerated if the score is > 0.05. Mutation Assessor output result «neutral» or «low» are predicted to not impact protein function, whereas variants classed as «medium» or «high» are predicted to result in altered protein function. N/A – result is not available. PolyPhen-2. A variant is appraised qualitatively, as «benign», «possibly damaging», or «probably damaging» based on pairs of false positive rate (FPR) thresholds, optimized separately for each model (HumDiv and HumVar). Probably damaging is more confident prediction, possibly damaging is less confident prediction, benign – no effect no protein structure and function. Total score is a total number of prediction algorithms which repot that given variant has an impact on protein function and structure. Score 0.5 was assigned for result «medium» by Mutation Assessor and different prediction results between HumDiv and HumVar.

**Supplementary Table 10.** Novel variants and coding SNPs with general MAF<0.05 detected in the patients by *CSF3R* DNA sequencing.

| Patient | Diagnosis | GCSF dosage, µg/kg/day, (SCT, yes/no) | Amino acid exchange | dbSNP | General MAF | Average heterozygosity | Genome position, chr1 | Reference nucleotide | Variant nucleotide | Position in GCSFR | Reference sequence |
| --- | --- | --- | --- | --- | --- | --- | --- | --- | --- | --- | --- |
| 0SU | CN | N/A |  |  |  |  |  |  |  |  |  |
| 0TQ | CML | N/A | p.E808K | rs146617729 | T=0.0020/10 | 0.015 +/- 0.084 | 36932047 | C | T | intracellular domain | NP_000751.1 |
| 09J | CN | 1.29 | p.M231T | rs3917973 | G=0.0260/130 | 0.046 +/- 0.144 | 36938269 | A | G | CRH domain | NP_000751.1 |
| 09J | CN | 1.29 | p.Q346R | rs3917974 | C=0.0260/130 | 0.046 +/- 0.144 | 36937701 | T | C | fibronectin type III like domain | NP_000751.1 |
| 09V | CN-MDS/AML | N/A |  |  |  |  |  |  |  |  |  |
| 028 | AiN | 0.2 |  |  |  |  |  |  |  |  |  |
| 0Q7 | CN | 3.93 |  |  |  |  |  |  |  |  |  |
| 0Q8 | CN | 3.4 |  |  |  |  |  |  |  |  |  |
| 0DL | CN | (11.3) SCT | p.D510H | rs3917991 | G=0.0557/279 | 0.096 +/- 0.197 | 36934805 | C | G | fibronectin type III like domain | NP_000751.1 |
| 0QI | CN | 1.63 |  |  |  |  |  |  |  |  |  |
| 0QL | CN | N/A |  |  |  |  |  |  |  |  |  |
| 0TB | de novo AML | no GCSF |  |  |  |  |  |  |  |  |  |
| 0WG | CN | N/A |  |  |  |  |  |  |  |  |  |
| 09V | CN-MDS/AML | N/A | p.G683R^1^ | rs3918001 | T=0.0495/248 | 0.080 +/- 0.183 | 36932503 | C | T | cytoplasmic | NP_724781.1 |
| 09A | CN | no GCSF |  |  |  |  |  |  |  |  |  |
| 093 | GSD/AML | 5 |  |  |  |  |  |  |  |  |  |
| 08Y | CN | 5.14 |  |  |  |  |  |  |  |  |  |
| 025 | AiN | N/A |  |  |  |  |  |  |  | CRH domain |  |
| 032 | SDS | no GCSF | p.D320N | rs3918018 | T=0.0100/50 | 0.029 +/- 0.117 | 36937878 | C | T | with WSXWS motif | NP_000751.1 |
| 0SR | CyN | 0.71 |  |  |  |  |  |  |  |  |  |
| 0SL | CN | N/A |  |  |  |  |  |  |  |  |  |
| 0U3 | CML | N/A |  |  |  |  |  |  |  |  |  |
| 0WJ | CN | N/A |  |  |  |  |  |  |  |  |  |
| 0RM | CyN | 1.87 | p.E405K | rs3918019 | T=0.0016/8 | 0.008 +/- 0.064 | 36937106 | C | T | fibronectin type III like domain | NP_000751.1 |
| 0SV | SDS | N/A | p.R440Q | rs3918020 | T=0.0024/12 | 0.007 +/- 0.059 | 36935408 | C | T | fibronectin type III like domain | NP_000751.1 |
| 0TB | SDS | SCT | p.M696T | rs148916169 | G=0.0012/6 | 0.006 +/- 0.053 | 36932463 | A | G | Cytoplasmic | NP_724781.1 |
| 0UD | SDS | (no G-CSF) SCT | p.E149D | rs139332126 | G=0.0012/5 | 0.006 +/- 0.053 | 36939403 | C | G | fibronectin type III like domain | NP_000751.1 |
| 0S3 | CyN | 1 | p.A750T | rs142939584 | NA | 0.000 +/- 0.015 | 36931801 | C | T | Cytoplasmic (splice-acceptor_1) | NP_758519.1 |
| 02F | CyN | no GCSF | p.M222T | novel | novel | novel | 36939044 | A | G | fibronectin type III like domain | NP_000751.1 |
| 09O | CN | no response to GCSF | p.W547* | rs138156467 | T=0.0002/1 | 0.001 +/- 0.021 | 36933759 | G | A | fibronectin type III like domain | NP_000751.1 |
| 09O | CN | no response to GCSF | c.998-2A>T | novel | novel | novel | 36937742 | A | T | - | NP_000751.1 |
| 0X6 | CN | 40 | p.R311C | rs201890478 | NA | 0.002+/-0.032 | 36937905 | G | A | CRH domain | NP_000751.1 |
| 0X6 | CN | 40 | p.Q114* | novel | novel | novel | 36940999 | G | A | Ig-like C2-type domain | NP_000751.1 |
|  | | | | | | | | | | | |

All 13 low frequency db SNPs (general MAF< ~0.05) and 3 novel variants identified in the studied patient’s group are listed. For each SNPs, if applicable, amino acid exchange, dbSNP number, general minor allele frequency (MAF), average heterozygosity, genome position, reference and variant nucleotide, localization in the G-CSFR domains, corresponding reference sequence are indicated. CN – severe congenital neutropenia, CyN – cyclic neutropenia, AiN – autoimmune neutropenia, CN-MDS/AML - CN patients who progressed to leukemia or MDS, GSD/AML – patient with glycogen storage disease Ib who progressed to AML, SDS – Shwachman-Diamond Syndrome, N/A – information regarding rhG-CSF dose is not available, CRH domain – cytokine receptor homology domain, no G-CSF – patient not treated with rhG-CSF, SCT – stem cell transplantation, *- stop codon. The position of the dbSNP on protein level is based on corresponding reference protein sequence (last column in the table). ^1^- translated in G-CSFR isoform III only.
